# Supplementary material for: Impact of Air Pollution and COVID-19 Infection on Periprocedural Death in Patients with Acute Coronary Syndrome
Source: Int J Environ Res Public Health. 2022 Dec 11;19(24):16654. doi: 10.3390/ijerph192416654 (PMC9778735; doi:10.3390/ijerph192416654)
Supplement: Supplementary file 1 [file ijerph-19-16654-s001.zip › ijerph-1990627-supplementary.pdf]

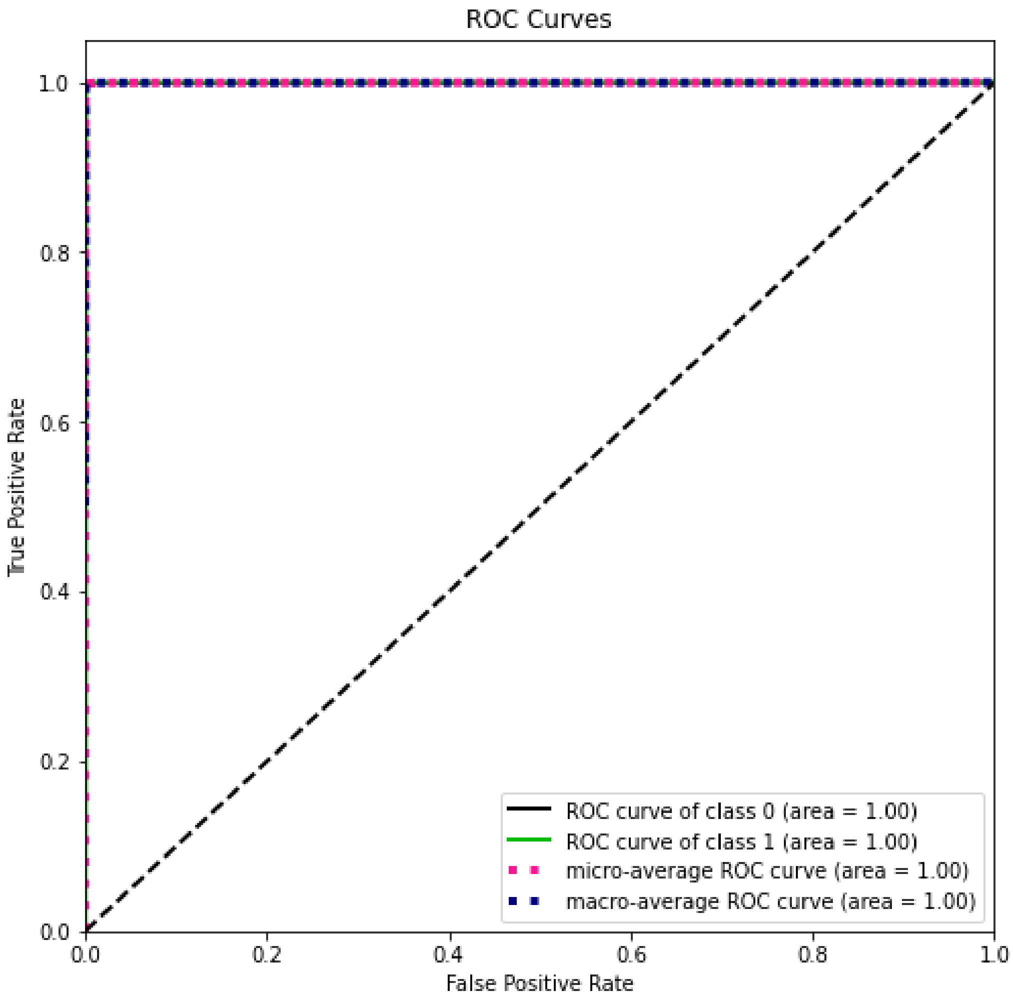

Figure S1. Evaluation of the validation phase of the model with the area under the receiver operating characteristics curve (AUCROC).

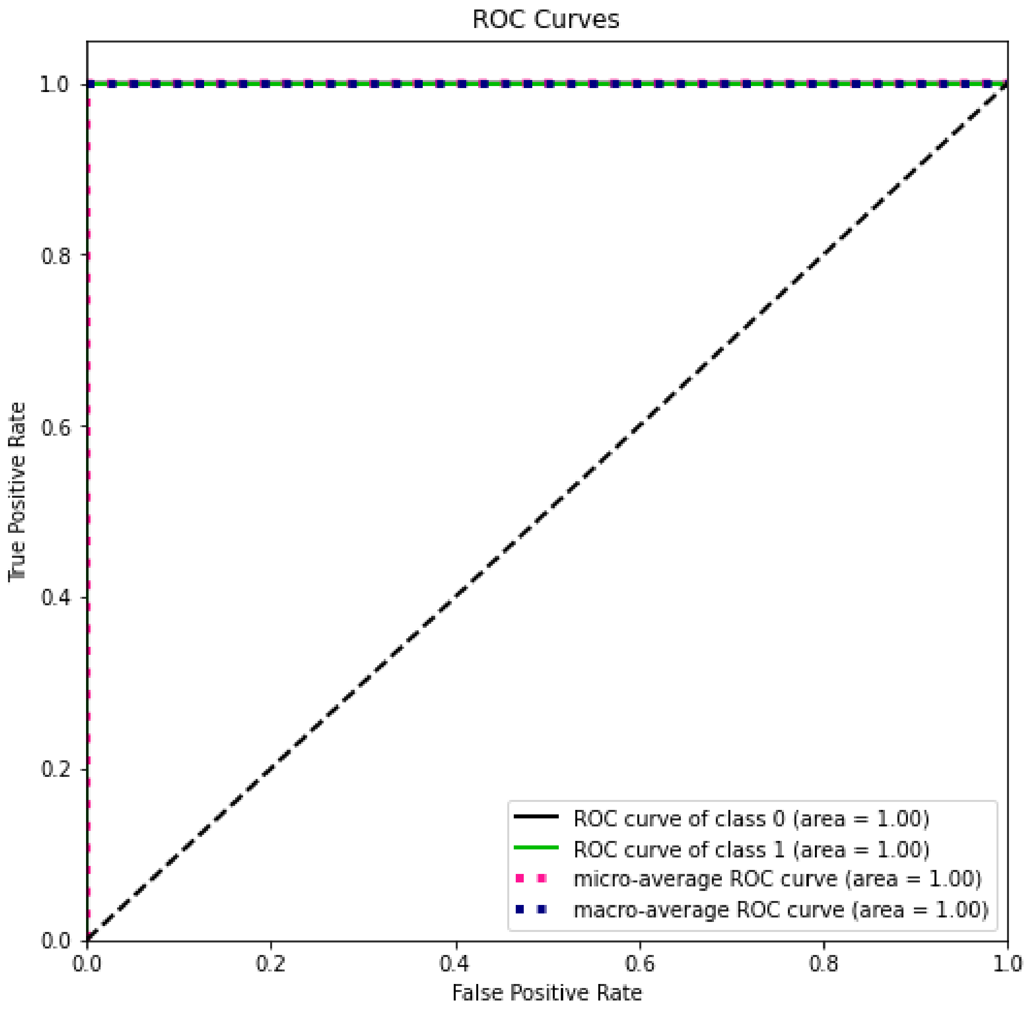

Figure S2. Evaluation of the training phase of the model with the area under the receiver operating characteristics curve (AUCROC).
